# New insights in Rett syndrome using pathway analysis for transcriptomics data

**DOI:** 10.1007/s10354-016-0488-4

**Published:** 2016-08-12

**Authors:** Friederike Ehrhart, Susan L. M. Coort, Elisa Cirillo, Eric Smeets, Chris T. Evelo, Leopold Curfs

**Affiliations:** 1 Department for Bioinformatics – BiGCaT, Maastricht University, Maastricht, The Netherlands; 2 Governor Kremers Centre - Rett Expertise Centre, Maastricht University Medical Centre, Maastricht, The Netherlands

**Keywords:** Rett syndrome, Rare disease, Systems biology, Pathway analysis, Bioinformatics, Rett-Syndrom, Seltene Krankheit, Systembiologie, Pathway-Analyse, Bioinformatik

## Abstract

The analysis of transcriptomics data is able to give an overview of cellular processes, but requires sophisticated bioinformatics tools and methods to identify the changes. Pathway analysis software, like PathVisio, captures the information about biological pathways from databases and brings this together with the experimental data to enable visualization and understanding of the underlying processes. Rett syndrome is a rare disease, but still one of the most abundant causes of intellectual disability in females. Cause of this neurological disorder is mutation of one single gene, the methyl-CpG-binding protein 2 (*MECP2*) gene. This gene is responsible for many steps in neuronal development and function. Although the genetic mutation and the clinical phenotype are well described, the molecular pathways linking them are not yet fully elucidated. In this study we demonstrate a workflow for the analysis of transcriptomics data to identify biological pathways and processes which are changed in a *Mecp2*^-/y^ mouse model.

**Electronic supplementary material:**

The online version of this article (doi: 10.1007/s10354-016-0488-4) contains supplementary material, which is available to authorized users.

## Zusammenfassung

Die Analyse von Transkriptomdaten ist in der Lage, ein Gesamtbild der veränderten zellulären Prozesse widerzugeben. Dafür bedarf es aber spezieller bioinformatischer Methoden, um diese Veränderungen sicher zu identifizieren. Mit einer Software, wie PathVisio z. B., wird auf eine Datenbank mit Informationen über biologische Stoffwechsel- und Signalwege zugegriffen und diese mit den experimentell ermittelten Daten abgeglichen. Damit können diese visualisiert und die zugrunde liegenden biologischen Prozesse besser verstanden werden. Das Rett-Syndrom ist eine seltene Krankheit und eine der häufigsten Ursachen für geistige Behinderung bei Frauen. Die Ursache sind Mutationen im *MECP2*-Gen („methyl-CpG-binding protein 2“), einem wichtigen epigenetischen Regulator in Nervenzellen. Zwar wurden die Mutationen und daraus resultierende klinische Phänotypen gut beschrieben, doch besteht noch kein umfassendes Verständnis der vernetzenden molekularen Prozesse. In der vorliegenden Studie stellen die Autoren einen Workflow für die Analyse von Transkriptomdaten dar, mit dem sich die veränderten biologischen Stoffwechsel- und Signalwege in einem *Mecp2*^-/y^-Maus-Modell identifizieren lassen.

## Introduction

**Fig. 1:**
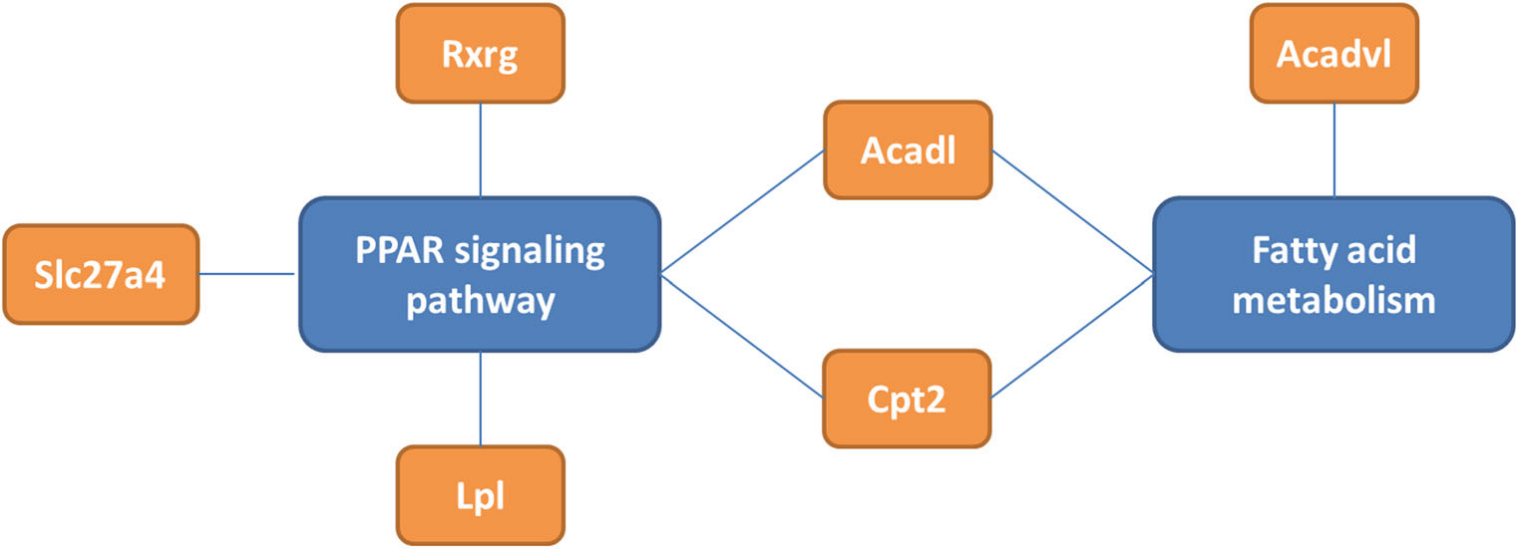
Demonstration of how gene ontology annotations lead to understanding of biological networks. The genes Slc27a, Lpl, Rxrg, Acadl, Cpt2, and Acadvl are differentially expressed in interneurons. All of them except Acadvl contribute to the peroxisome proliferator-activated receptor (PPAR) signaling pathway which is therefore assumed to be changed. Three of these genes, Acadl, Acadvl, and Cpt2 are also involved in fatty acid metabolism. The overlapping genes of the PPAR signaling pathway and fatty acid metabolism are Acadl and Cpt2. The number of direct connections can be used as an indicator how well connected—how important—a process or a pathway is. Using this existing knowledge in combination with experimental data, it is possible to analyze, visualize, and understand a network of changed processes

Progress in biomedical methods today enables generation of vast amounts of molecular data, namely transcriptomics data with up to 100,000 transcripts which occur in a human cell. The current challenge is to identify the needle of changed processes in the haystack of data, and to combine different types of data. Pathway analysis combines these experimental transcriptome data with structured existing knowledge. Bioinformatics analysis and visualization methods and tools allow explanation and proof of whether and how a certain biological pathway is altered as a consequence of the experiment, which may be a mutation, drug application, nutrition, or disease. One of the strengths of this approach is that it provides a general picture of how the biological network is affected without being biased by hypothesis. Instead of hypothesis-driven experiments and data analysis, we are speaking of data-driven approaches [1]. There are databases for single entities like genes, transcripts, proteins, and metabolites (e. g. Ensembl, NCBI, MSeqDR, UniProt, ChEBI) [2], as well as repositories of structured biological knowledge like WikiPathways.org [3], which is a curated database of biological pathways. WikiPathways.org contains biological pathways of several species, namely metabolite–enzyme interactions, gene signaling cascades, receptor–target interactions, and disease-related pathways, and can be used for reviewing and visualizing knowledge [4] or data analysis (and visualization) [1, 5–7] (http://wikipathways.tumblr.com/). Gene annotations, as defined in gene ontology [8], give machine-readable information about the cellular component in which this gene is present (e. g., nucleus, mitochondria), the molecular function (e. g., membrane-binding or esterase activity), and the biological process (e. g., negative regulation of neuronal differentiation). Single genes can contribute to multiple processes and link pathways with each other. Fig. 1 shows an example how gene annotations lead to understanding of biological network changes, e. g., fatty acid metabolism in MECP2-deficient mouse interneurons.

If a group of genes which have the same or linked gene ontology annotation are up- or downregulated together, we speak of term enrichment or overrepresentation, which is measurable and gives information about the functions, processes, and cellular components that are changed. This approach is especially useful for investigation of rare diseases and disorders like Rett syndrome, where the genetic cause and the clinical phenotypes are well known, but there are still gaps in understanding how the biological pathways are modified and lead to these phenotypes.

Rett syndrome is a rare disorder which was first described by Andreas Rett [9] and affects about 1:10,000 females [10]. Typical symptoms are regression and loss of acquired skills at the age of 6–18 months after a normal pre- and postnatal development. Rett females are typically severely intellectual disabled, hardly able to perform directed movements (including gait and speech), although stereotypic repetitive movements (“hand washing”) are often observed. Typical additional symptoms are abnormal cardiac and respiratory function, scoliosis, epilepsy, and sleep problems [11]. The cause of this disorder is a mutation—de novo in almost all cases—in the gene for methyl-CpG-binding protein 2 (*MECP2*), which is located on the X chromosome [12]. Rett females are typically heterozygous while hemizygous males are usually not viable. There are about 200 different mutations known which lead to Rett syndrome. These occur mostly in either the DNA binding domain—a loss in specificity to recognize and bind methylated DNA is already enough to lead to Rett syndrome [13]—or the transcription repressor binding domain.

The objective of this study is to present a bioinformatics workflow to give an overview of changed biological pathways in Rett syndrome using transcriptomics data. In this study we investigated the differentially expressed genes and affected pathways in four different primary neuronal cell types using published (but not investigated to this extent) data from a *Mecp2*^-/y^ mouse model originally published by Sugino et al. [14].

## Materials and methods

### The data source

In the current study a previously published study from Sugino et al. [14] was used. These authors investigated a special mouse model which was bred from GFP-/YFP-labeled transgenic G42 and TH line, crossed with mice lacking functional Mecp2 and backcrossed with C57BL/6. They selected male, 37–55-day-old, hemizygous null (*Mecp2*^-/y^) or wildtype controls. Brain tissue slices of the desired area were cut out and cell sorting was performed: 1. layer 5 thick tufted pyramidal neurons (TTL5) in the motor cortex labeled in YFPH line, 2. fast-spiking parvalbumin-positive interneurons in the motor cortex labeled in the g42 line, 3. noradrenergic locus coeruleus neurons labeled in the TH line, and 4. cerebellar Purkinje cells labeled in the G42 line. Three samples, each from a different animal, were taken for microarray analysis. After extraction and preparation, the RNA was hybridized to Affymetrix Mouse Genome 430 2.0 oligonucleotide arrays (MOE430v2) (Affymetrix, Santa Clara, USA) [14]. This dataset was uploaded and published on ArrayExpress (E-GEOD-8720) [15].

### Data quality control, pre-processing, and statistics

In ArrayAnalysis.org, namely the AffyQC and statistics tool [16], the quality of the selected raw transcriptomics data was controlled, the data were normalized using GC-RMA, and statistically analyzed using an adapted *t*-test. Finally, a list of significantly changed genes for each cell type (e. g., Purkinje cells of *Mecp2*^-/y^ vs. wild type) was obtained using a cutoff of a ±0.5 log2-fold change and *p* ≤ 0.05.

### Pathway analysis

For pathway analysis we used PathVisio (version 3.2.0) [17]. The pathway repository used to perform pathway statistics was WikiPathways [3], namely the curated collection of *Mus musculus*-specific pathways (28.4.2016). Data integration for the variety of identifiers requires use of BridgeDb identifier mapping service [18]. To calculate z‑score—an index representing whether and how much a pathway is changed if pathway components are changed—and *p*-values, the following logic was used: ((LogFC ≤ -0.5 OR LogFC ≥ 0.5) AND *p* ≤ 0.05). The pathways which had z‑score >1.96, minimum number of changed genes >3, and *p* ≤ 0.05 were considered as changed.

### Gene ontology term enrichment analysis

Gene ontology term enrichment analysis was performed using GO-Elite (version 1.2.5) [19]. We compared the list of significantly changed genes versus all investigated genes. The number of permutations for overrepresentation analysis was set to 2000, z‑score to >1.96, *p* ≤ 0.05, and minimum number of changed genes ≥3. Only Ensembl annotations were taken into the analysis, which means not considering 64 Affymetrix IDs out of 17,417 identifiers in the list of all genes investigated. Results of this analysis were visualized using Cytoscape (version 3.4.0) [20] and the number of direct neighbors of a data node was used as an indicator. The pathway and network analysis workflow was described previously [1].

## Results

The original study by Sugino et al. already indicated that each of the four investigated subpopulations of neuronal cells of a *Mecp2*^-/y^ mouse model expresses a different set of genes in comparison to wildtype, and they found especially cell adhesion genes and long genes (>20 kbp) to be overrepresented among the changed genes. In the present study, the raw transcriptomics data were reanalyzed using ArrayAnalysis.org (the quality control, QC, report can be found in the supplementary data). Finally, lists of differentially expressed genes were extracted (supplementary table 1) and pathway and gene ontology term enrichment analysis was performed to reveal the biological pathways changed in this model system. 258 genes are differentially expressed in Purkinje cells, 850 in locus coeruleus neurons, 463 in TTL5, and 301 in fast-spiking interneurons.

### Pathway analysis

**Fig. 2:**
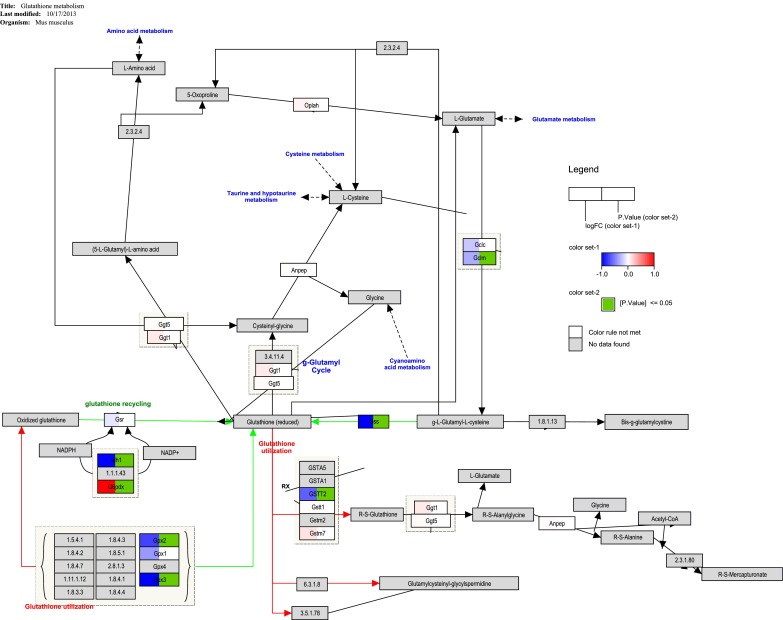
Pathway visualization of changed glutathione pathway in locus coeruleus neurons. Glutathione pathway is from WikiPathways (http://www.wikipathways.org/instance/WP164) supplemented with the experimental data. Blue boxes indicate downregulation, red upregulation of this gene and a green marker in the box indicates that this change is significant (*p* ≤ 0.05). Note that several genes which catalyze the reaction from glutamate to glutathione are downregulated

In each cell type there are different sets of pathways affected (supplementary table 2). Fatty acid metabolism (fatty acid oxidation, mitochondrial fatty acid beta-oxidation, and adipogenesis) are the predominantly changed pathways in fast-spiking interneurons of the motor cortex (Fig. 1). The expression of Gcdh (glutaryl-coenzyme A dehydrogenase), Lpl (lipoprotein lipase), Acadl (acyl-coenzyme A dehydrogenase, long chain), Acadvl (acyl-coenzyme A dehydrogenase, very long chain), and Slc25a20 (solute carrier family 25 [mitochondrial carnitine/acylcarnitine translocase], member 20) is down, whereas the expression of Cpt2 (carnitine palmitoyltransferase 2) and Chkb (choline kinase beta) is upregulated. In the locus coeruleus neurons, glutathione and amino acid metabolism is affected, in which the expression of glutathione-producing enzymes is significantly downregulated (Fig. 2). Gss (glutathione synthetase) and Gclm (glutamate-cysteine ligase, modifier subunit), both enzymes which are directly involved in the production of glutathione out of glutamate, are significantly downregulated. Supplementary table 2 gives a complete overview of the changed biological pathways in the four cell types of * Mecp2*^-/y^ mice compared to wildtype mice.

### Gene ontology term enrichment analysis

Gene ontology in general gives a triplet of annotation (information) for each gene: cell compartment (localization), molecular function, and biological process in which this gene is involved. Term enrichment analysis gives information about whether these terms are over- or underrepresented for a certain dataset, and, therefore, whether these biological processes, molecular functions, or cellular compartments are especially affected. The connections between these single changed processes were linked and visualized using Cytoscape. The visualization of this network allows determination of which differentially expressed genes are interfering with different processes—and whether they are linking several processes with one another.

The affected cellular compartment was mainly the nucleus and nucleus-related processes like the DNA-bending complex or regulation of histone modification, except for the TTL5 (supplementary table 3). Cell adhesion- and cytoskeleton-related processes (e. g., actin filament, filopodium) are changed in all cell types except for the fast-spiking interneurons. Neurospecific functions (e. g., postsynaptic membrane, neuron projection) were found to be changed in all four cell types investigated.

Investigating molecular function, glutamate binding and glutamate receptor activity was changed in Purkinje cells and TTL5; while ion flux-related receptors, channels, and binding proteins changed in all cell types (supplementary table 3). The specific ions were mostly calcium and potassium, indicating neuronal function and signal transduction. Receptor activities for IGF, androgen, semaphorin, ephrin, β‑tubulin, actin, clathrin, and steroid hormones were also affected. Locus coeruleus neurons revealed a lot of changed enzyme activity.

**Fig. 3:**
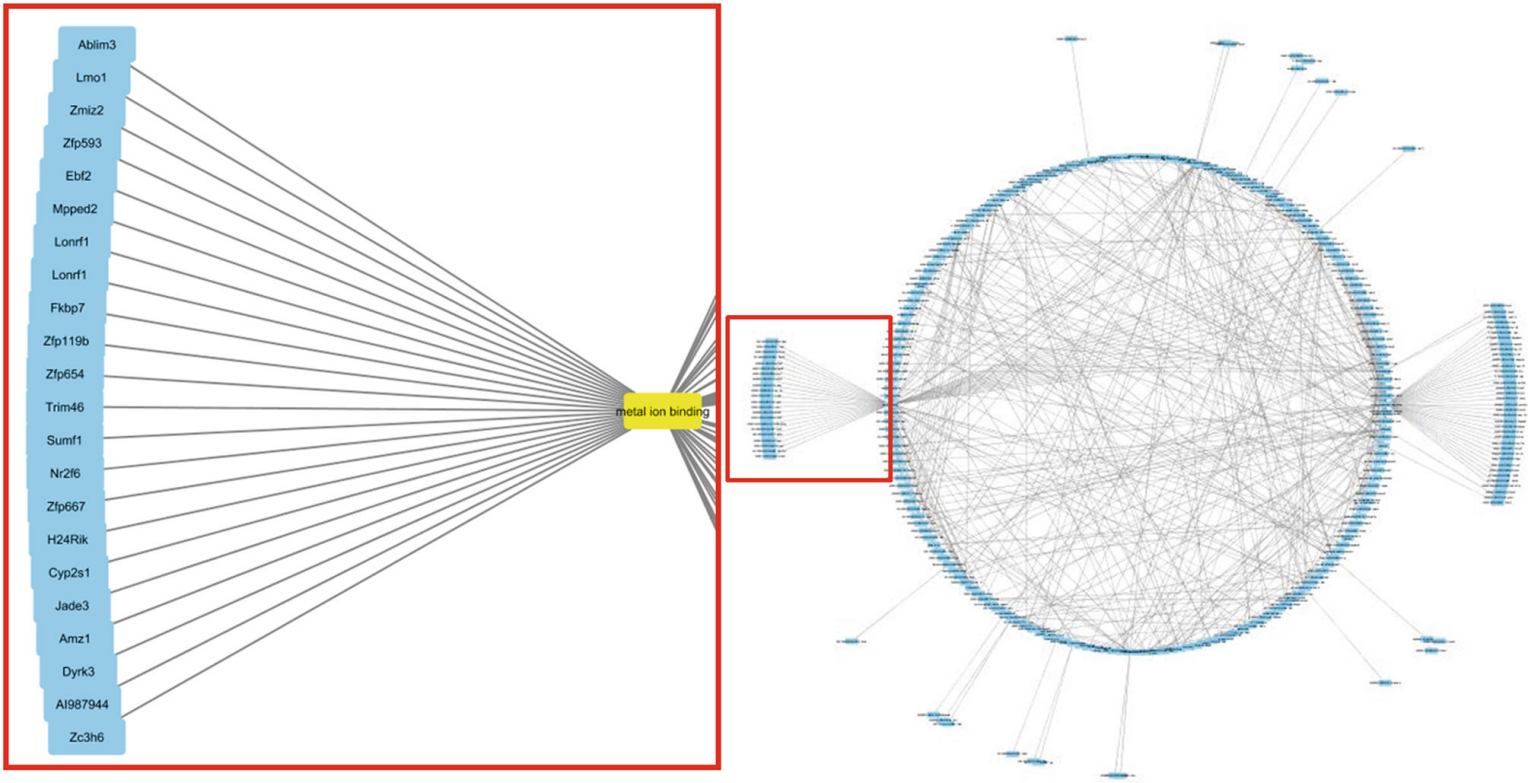
Network visualization of changed processes in Purkinje cells of *Mecp2* ^-/y^ vs. wildtype using Cytoscape [20]. Highlighted is the biological process of metal ion binding with the genes which are differentially expressed

**Fig. 4:**
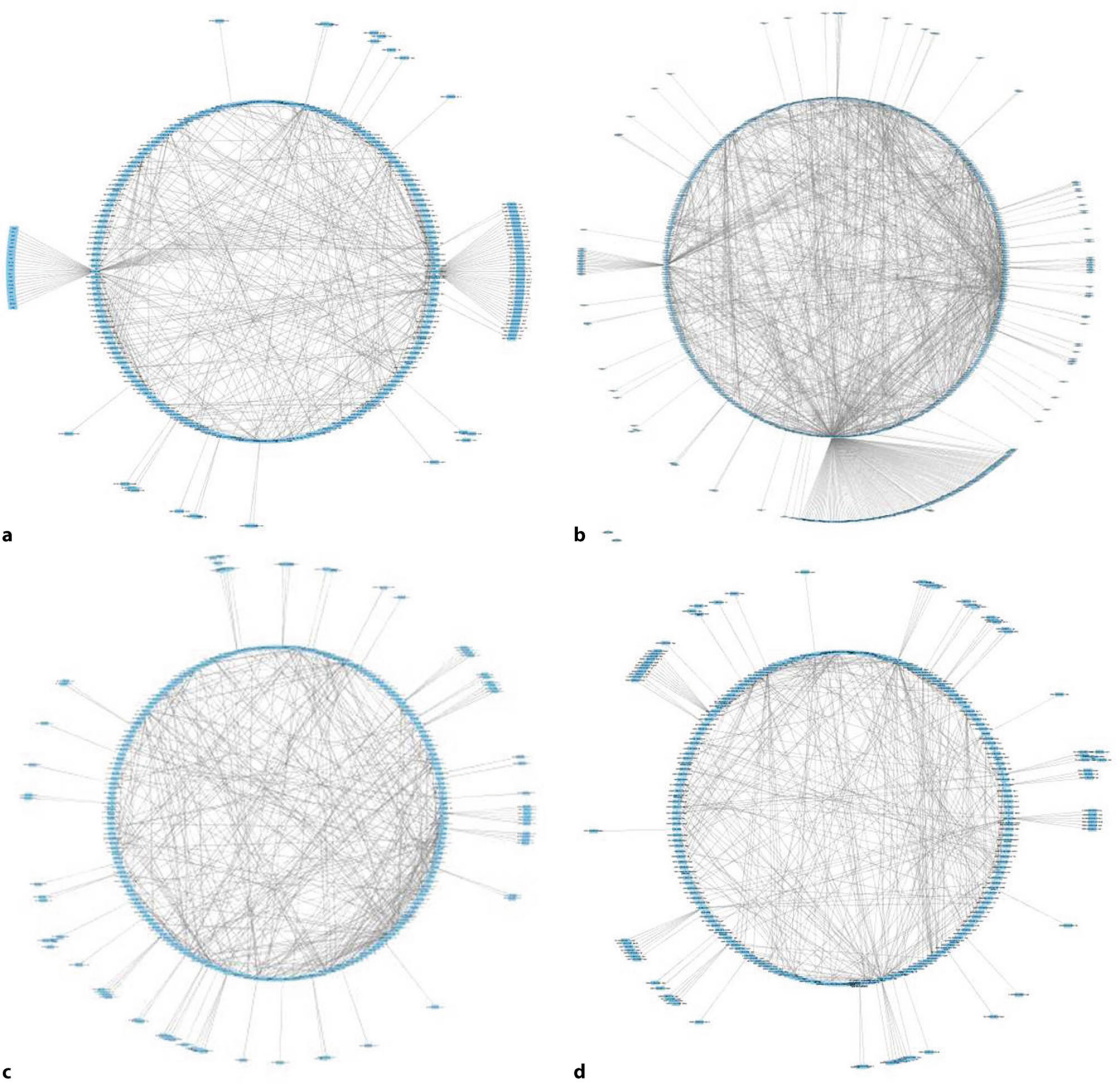
Network visualization of changed processes in **a** Purkinje cells, **b** locus coeruleus neurons, **c** TTL5, **d** fast-spiking interneurons. High-resolution images of these networks are in the supplementary data

There are between 46 and 165 biological processes changed for each cell type (supplementary table 3). For Purkinje cells, the most changed processes (highest z‑score, indicating the chance that there are enough differently expressed genes to affect the whole process) are semicircular canal morphogenesis, regulation of cholesterol metabolic process, and behavioral defense response. The network analysis revealed the affected biological processes that are most connected with other processes, which are metal ion binding and intracellular signal transduction. Fig. 3 shows the visualization of changed processes in Purkinje cells and especially the genes/proteins annotated with metal ion binding which are differentially expressed. Fig. 4 shows the changed processes for all cell types. The high resolution images are available in the supplementary material.

Locus coeruleus neurons: With the highest z‑scores are the processes sympathetic ganglion development, self-proteolysis, and detection of temperature stimulus. Network analysis showed negative regulation of cell proliferation and regulation of neuron projection to be the most connected processes.

TTL5: Behavioral defense response, labyrinthine layer blood vessel development, pyrimidine-containing compound catabolic process, and negative regulation of BMP signaling pathway are the biological processes changed with the highest z‑scores. The most connected processes are regulation of ion transport and regulation of neuron differentiation.

Fast-spiking interneurons: The highest z‑scores are glutamate binding, myelin sheath, regulation of cholesterol metabolic process, and axon development. The most connected processes are regulation of neuron differentiation and regulation of cell projection organization.

## Discussion

The present study is a data-driven approach to investigate the changes in gene expression and combine the results with prior knowledge. The transcriptomics data were obtained from a previously published study by Sugino et al. [14] using four different subpopulations of neuronal cells in a *Mecp2*^-/y^ mouse model. We reanalyzed the raw transcriptomics data and applied pathway and gene ontology analysis. The analysis confirmed the change of biological processes and functions belonging to neuro-specific metabolism: especially synapsis function [21] and glutamate/glutathione metabolism [21–23] are affected in *Mecp2*^-/y^ mice, and a mismatch between excitatory and inhibitory neuronal activities is observed [21, 24]. These are known to be changed in Rett syndrome (in humans and in a mouse model) and contribute to disorder development and etiology. New insights are found by looking at the specific changes of individual cell types, indicating that for disorder development and symptoms, the different neuronal cell populations are differentially affected.

- Purkinje cells are GABAergic neurons. They are inhibitory, highly connected (about 500 synapses each), and contribute to motoric coordination. Changes in ion transport and membrane trafficking highly affects their function. For Rett syndrome, the mismatch between inhibitory and excitatory neuronal pathways are known contribute to disorder development [24].
- Locus coeruleus neurons: the locus coeruleus is located in the brainstem and is one of the main producers of norepinephrine. It is furthermore related to several neuronal functions like sleep/wake cycle, posture and balance, and neuroplasticity, which are indeed impaired in Rett females. It is also the center which is affected in several other neurological disorders like Parkinson’s disease. An impaired regulation of cell proliferation (which may be a cause or a consequence of changed amino acid pathway) and affected neuronal differentiation affects its proper function.
- TTL5: TTL5 are excitatory neurons and most important for information processing. They are the neurons with the most extensive arborization and connect the different cortical layers. Also here is ion transport most relevant for neurological signal transduction, and proper regulation of excitatory and inhibitory signals is crucial for function.
- Fast-spiking interneurons: interneurons are involved in positive and negative feedback mechanisms, they are able to transduce excitatory to inhibitory signals, and can create (or contribute) to electric oscillations in neuronal networks. Fatty acid metabolism was found to be affected especially here (Fig. 1). The metabolomics study of Viola et al. found different levels of certain phospholipids in a Rett syndrome mouse model, but it is not clear yet whether this is cause or consequence of reduced cell growth [22].

The present study shows how pathway and gene ontology analysis tools can be used to find biological processes of interest based on changes in gene expression. A more detailed investigation of the data is necessary, especially to separate between up- and downregulated processes, and to link them to their specific function. Knowing the changed processes, and the single genes and proteins behind these processes, furthermore allows searching for specific drugs which bind and modify these targets. We expect that the application of these tools and methods is going to improve not only research on Rett syndrome or other neurological disorders, but will contribute to a better understanding of neurological physiology in general.

## Caption Electronic Supplementary Material

Table 1-3
